# Social support moderates stress effects on depression

**DOI:** 10.1186/1752-4458-8-41

**Published:** 2014-11-13

**Authors:** Xingmin Wang, Lin Cai, Jing Qian, Jiaxi Peng

**Affiliations:** Anhui Provincial Committee Party School, Hefei, China; Department of International Exchange and Cooperation, Kunming University, Kunming, China; School of Business, Beijing Normal University, Beijing, China; Department of Psychology, Fourth Military Medical University, Xi’an, China

**Keywords:** Stress, Social support, Depression, Moderating effect, University students

## Abstract

This study examined the moderator effect of social support on the relationship between stress and depression of university students. A total of 632 undergraduate students completed the measures of perceived stress, perceived social support, and depression. Hierarchical regression analysis showed that social support moderated the association between stress and depression. Undergraduate students with high stress reported higher scores in depression than those with low stress with low social support level. However, the impact of stress on depression was much smaller in the high social support group compared with that in the low social support group.

## Introduction

Studies that focus on college students’ mental health problems are gradually increasing because of significant increase in the incidence of mental disorder among college students [1–3]. Depression is a common mental disorder mainly characterized by significant and constant down in spirits [4]. Emotional depression varies from moodiness to grief, low self-esteem, depression, and even pessimism, which may lead to suicidal attempt or behavior [5, 6]. Previous studies have found that depressive symptoms are widespread in college students [7]. Surveys have found that the Chinese mainland college student depression rate is 15% to 35% [8–10].

Stress, a mental experience caused by demand and failure, is very common in our lives [5]. However, stress may further cause negative emotions, such as depression and anxiety, and may even hinder normal development of the personality and behavior of a person if not properly controlled and responded to [11]. Depression is created through interaction of various factors, including environmental and individual factors [12]. After summarizing the results obtained from nearly twenty years of research about the relationship between stress and depression, Kessler indicated that stress is closely related with depression and stress intensity and degree of depression have a dose-response relationship [13, 14]. However, not all people will have depression when under pressure. The depression degree of different individuals varies even under the same stress conditions [15, 16], indicating that other variables affect the relationship between stress and depression.

Social support is the care or help from others that an individual can feel, notice, or accept [17, 18]. As an important environmental resource in an individual’s social life, social support affects a person’s physical and mental health and behavior patterns, and has a very close relationship with the generation, development, control, and prevention of depression [19–21]. A good social support can provide protection for an individual under stress and has common gaining function on maintaining an individual’s good emotional experience [22].

Existing studies on graduate student groups have focused on mutual relationship among stress, social support, and depression. We consider that the mental mechanism research on depression generation should deeply discuss the cooperation and antagonism between different factors. Based on previous studies, we assume that relationship between stress and depression is affected by social support, a moderating variable between stress and depression.

## Methods

### Participants and procedure

The participants included 632 undergraduate students comprising 315 women and 317 men from Chonging City, China. The ages of the participants ranged from 18 to 22 years old, with a mean of 20.47 (SD = 1.74). The participants received ¥15 as compensation. A total of 632 scales were distributed and collected; all scales were valid. All participants provided their written informed consent before completing the measures.

### Instruments

#### Perceived stress scale

The Perceived Stress Scale is a self-report instrument that evaluates the level of perceived stress during the past month and consists of 14 items with a 5-point response scale (0 = never, 1 = almost never, 2 = once in a while, 3 = often, 4 = very often). Higher score indicates higher level of perceived stress [23]. In this study, the Cronbach alpha coefficient for Perceived stress Scale was 0.850.

#### Perceived social support scale (PSSS)

PSSS, developed by Zimet et al., is a 12-item self-report measure of how an individual perceives the social support system, including an individual’s sources of social support (i.e., family, friends, and significant other). Items are rated from 1 (very strongly disagree) to 7 (very strongly agree) [24]. Three subscale scores for PSSS can be computed, namely, support from family, friends, and significant other. Examples of the items from this form are as follows: “I get the emotional help and support I need from my family” and “My friends really try to help me”. PSSS was translated by Chou [25] and has been proven to have good validity and reliability for the Chinese population (e.g., [26]). In the present study, the Cronbach alpha coefficient for PSSS was 0.848.

#### Self-rating depression scale (SDS)

SDS, developed by Zung [27], is a self-report measure of depression consisting of 20 items, with a four-point scale ranging from (1) a little of the time to (4) most of the time. Among the 20 items, 10 are worded positively and 10 are worded negatively. The former 10 items are reversed items. The validity and reliability of the SDS have been reported [27]. The researcher translated the 20-item version of SDS into Chinese, and this version has been proven to have good validity and reliability [28]. In the current study, the Cronbach alpha coefficient for SDS was 0.886.

## Results

### Bivariate analyses

The means, standard deviations, and inter correlations for each questionnaire are presented in Table 1. According to the SDS norm of Chinese college students, the total SDS score was greater than or equal to 41, which is deemed as the bound of depression [29]. About 18.7% of the participants had depression. Table 1 shows that stress is negatively correlated with social support and both stress and social support are correlated with depression.

**Table 1:** **Means, standard deviations, and correlations of stress, social support and depression**

	Mean	SD	1	2	3
1. Stress	18.44	7.35	1		
2. Social support	69.12	10.14	-0.53^**^	1	
3. Depression	11.84	11.84	0.44^**^	-0.45^**^	1

Not: _**_, p < 0.01.

### Test of the moderation model of social support

Hierarchical regression procedures were performed to test the moderating effect of social support on the relationship of stress and depression [30]. The social support and stress variables were standardized before testing for moderating effect to reduce problems related to multicolinearity between the interaction term and the main effects [31]. Thus, the z-scores were calculated for social support and stress. The order of entry in the hierarchical regression model was as follows. At step 1, the predictor variable (stress) was entered into the regression equation. At step 2, the moderator variable (social support) was entered into the regression equation. At step 3, the interaction of stress × social support was added. The significant change in R^2^ for the interaction term indicates significant moderator effect. The results of these analyses are presented in Table 2. Stress (β = 0.44, p < 0.01) significantly predicted depression in the second step. The social support factor added a significant increment to the model in step three, where social support (β = -0.30, p < 0.01) was significantly related to depression after controlling other variables. Moreover, a significant interaction between stress and social support (β = -0.10, p < 0.01) was present, as predicted. These findings suggest that social support moderated the impact of stress on depression.

**Table 2:** **Hierarchical regression analysis predicting depression from social support and stress**

	B	SE	***β***	***t***	***F***	R ^2^	△R ^2^
Step 1							
Stress	5.16	0.43	0.44	12.16	147.89	0.189	0.189
Step 2							
Social support	-3.59	0.48	-0.30	-7.50	56.24	0.254	0.066
Step 3							
Stress × Social support	-0. 84	0.31	-0.10	2.84	10.49	0.265	0.011

**Figure 1:**
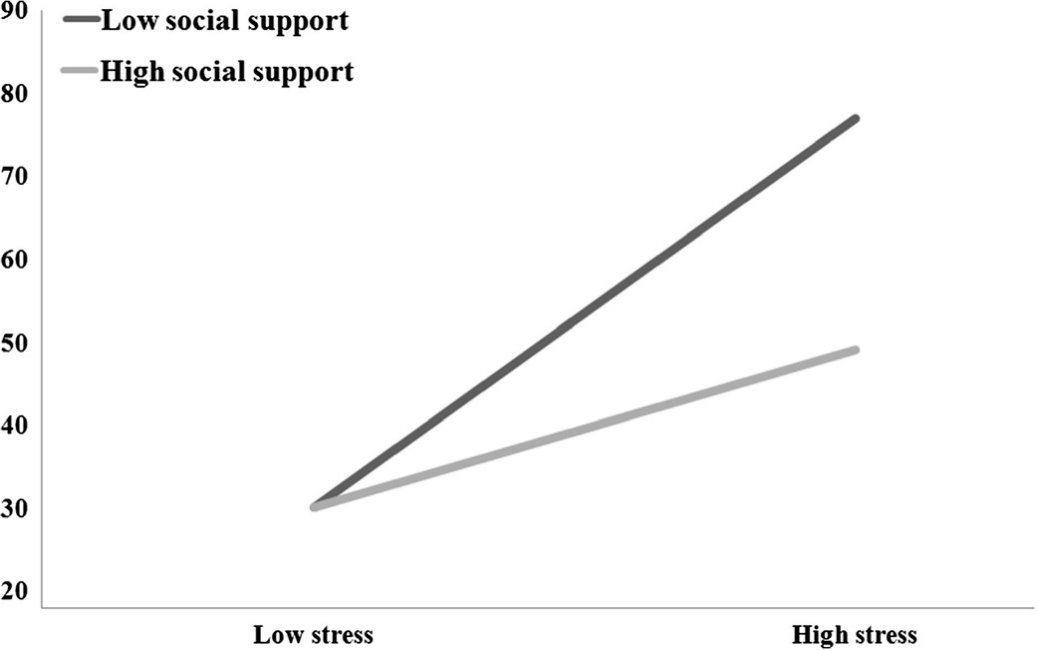
**The moderating effect of social support in the relationship between stress and depression.**

To illustrate the stress × social support interaction for depression, we plotted the regression of depression on stress at high and low levels of social support (see Figure 1). Consistent with the procedures outlined by Kong, Zhao, and You [32], we utilized a simple slope formula for regression of stress on depression using the high (above the mean) and low (below the mean) values for social support [32]. Figure 1 shows a significantly positive relationship between stress and depression at low levels of social support (β = 0.47, p < 0.01, R^2^ = 0.22). The relationship between stress and depression was significant at high levels of social support (β = 0.22, p < 0.01, R^2^ = 0.04). The regression coefficient, however, was significantly less than that in the low social support group. Hence, the results also suggest that social support moderated the impact of stress on depression.

### Structural equation modeling analysis

**Figure 2:**
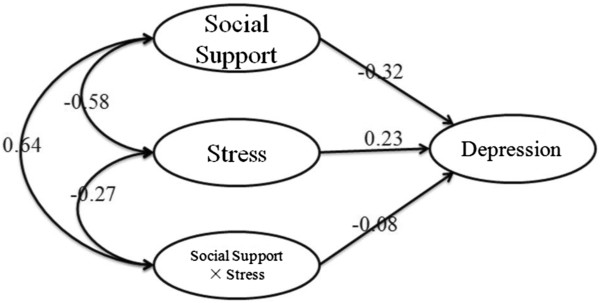
**The finalized structural model.**

The SEM unconstrained approach put forward by Marsh, Wen, and Hau was used to test the moderating role of organizational identification on the relationships between organizational justice and job satisfaction [32]. Confirmatory factor analysis was used to assess whether the measurement model adequately fit the sample data. A test of the measurement model yielded satisfactory fit to the data: χ^2^/df = 2.16, RMSEA = 0.071, SRMR = 0.046 and CFI = 0.974. All factor loadings of the indicators of the latent variables were significant (p < 0.001), indicating that all these latent constructs were well represented by their indicators. Thus, a structural model was built (Figure 2). The final moderating model yielded satisfactory fit to the data: χ^2^/df = 1.61, RMSEA = 0.031, SRMR = 0.015 and CFI = 0.997.

## Discussion

According to the SDS norm standard of Chinese college students, the total SDS score was greater than or equal to 41 points, which is deemed as the bound of depression [29]. The detection rate of people with depression was 18.7%, demonstrating that the incidence of depression among the college students is rather high and should be given more attention [9].

The correlation analysis in this research showed that graduate students depression has a significant positive correlation with stress, indicating that stress is an important factor that affects depression generation. The same results were found in previous researches about stress and depression regarding different groups as research objects [15, 33, 34]. College students are carrying higher expectations from society, family, and individual, and are suffering from various stresses including study, occupation, marriage, and family [35, 36]. The stresses caused by negative life events may make students depressed, such as heavy burden of study, growing occupation or entrance stress, failure in love, and dispute or misunderstanding with classmates [37–39].

This research found that relationship between stress and depression is affected by social support, compared with the team of high social support. A closer relationship was found between stress and depression in the low social support team. Social support plays a significant regulating effect on the relationship between stress and depression and is an important environmental resource [20, 40]. An individual’s good social support network allows him/her to gain self-esteem and self-efficacy easier, thereby resisting the generation of negative emotions such as depression [41–43]. When an individual is under stress, social support makes him/her underestimate the hazards and the verities of stress by enhancing their coping capacities perceived. Social support can also provide problem solving strategies to the individual, reduce the importance of the problem, and alleviate the harmful effects of stress experience [42, 44, 45]. These effects can reduce the intensity of the relationship between stress and depression, thereby lowering the degree and generation of depression.

The results of this research suggest that the high depressive symptoms among college students should be brought to the attention of relevant departments. To prevent college student depression, relevant departments should both optimize the environment of college student study and life, try to decrease the generation of negative life events, provide adequate social support for college students, and enhance their cognitive and coping capacities to improve their mental qualities.
